# Effect and Safety of Adding Metformin to Insulin Therapy in Treating Adolescents With Type 1 Diabetes Mellitus: An Updated Meta-Analysis of 10 Randomized Controlled Trials

**DOI:** 10.3389/fendo.2022.878585

**Published:** 2022-05-30

**Authors:** Ying Liu, Hongbo Chen, Hui Li, Liman Li, Jin Wu, Hong Li

**Affiliations:** ^1^ Department of Pediatrics, West China Second Hospital, Sichuan University, Chengdu, China; ^2^ Center of Translational Medicine, Key Laboratory of Birth Defects and Related Diseases of Women and Children of Ministry of Education, West China Second University Hospital, Sichuan University, Chengdu, China; ^3^ Key Laboratory of Birth Defects and Related Diseases of Women and Children (Sichuan University), Ministry of Education, Chengdu, China

**Keywords:** type 1 diabetes mellitus, adolescent, insulin, metformin, meta-analysis

## Abstract

**Background:**

The role of metformin in the treatment of adolescents with type 1 diabetes mellitus (T1DM) remains controversial. We conducted this updated meta-analysis to generate a comprehensive assessment regarding the effect and safety of metformin in treating adolescents with T1DM.

**Methods:**

We systematically searched PubMed, Embase, and the Cochrane Central Registry of Controlled Trials (CENTRAL) from their inception to November 2021 to identify randomized controlled trials evaluating the efficacy of metformin in the treatment of adolescents with T1DM. The primary outcome was the HbA1c level, and secondary outcomes included the body mass index (BMI), total insulin daily dose (TIDD) (unit/kg/d), hypoglycemia events, diabetes ketoacidosis (DKA) events, and gastrointestinal adverse events (GIAEs). Statistical analysis was conducted using RevMan 5.4 and STATA 14.0.

**Results:**

Ten studies enrolling 539 T1DM adolescents were included. Results suggested that metformin significantly decreased the HbA1c level at 12 months (mean difference [MD])=-0.50, 95% confidence interval [CI]=-0.61 to -0.39, P < 0.01); BMI (kg/m^2^) at 3 months (MD=-1.05, 95%CI=-2.05 to -0.05, P=0.04); BMI z-score at 6 months (MD=-0.10, 95%CI=-0.14 to -0.06, P<0.01); and TIDD at 3 (MD=-0.13, 95%CI=-0.20 to -0.06, P<0.01), 6 (MD=-0.18, 95%CI=-0.25 to -0.11, P<0.01), and 12 (MD=-0.42, 95%CI=-0.49 to -0.35, P<0.01) months but significantly increased the risk of hypoglycemia events (risk ratio [RR]=3.13, 95%CI=1.05 to 9.32, P=0.04) and GIAEs (RR=1.64, 95%CI=1.28 to 2.10, P<0.01). For remaining outcomes at other time points, no statistical difference was identified. Sensitivity analysis confirmed the robustness of all pooled results.

**Conclusions:**

The use of metformin might result in decreased BMI (kg/m^2^), BMI z-score, and TIDD and increased risk of hypoglycemia events and GIAEs in adolescents with T1DM. However, future studies are required to further confirm the optimal dose and duration of metformin therapy.

## Introduction

According to the issued statistics, as one subtype of diabetes mellitus (DM), type 1 DM (T1DM) accounts for approximately 5%–10% of all DM individuals, and most importantly, T1DM accounts for 90% of all diabetes in childhood (1). For patients with T1DM, intensive diabetes management with insulin therapy has been considered as the standard strategy for maintaining glycemic control near the normal range (2). Studies demonstrated a strong association between T1DM and the risk of cardiovascular disease (CVD) (3), and maintaining glycemic control has also been found to reduce the risk of microvascular complications and cardiovascular morbidity, although an increased risk of hypoglycemia was reported (4).

It is a regret that the therapeutic goal proposed by the American Diabetes Association was not achieved in most patients with T1DM (5). Accurately speaking, the glycemic target was achieved in only 14% of children with T1DM when a criterion of HbA1c <7.5% was set (6). Certainly, another important fact is that intensive insulin therapy can result in substantial weight gain, which adversely increases the risk of CVDs (7). Therefore, it is essential to develop some novel adjunct non-insulin therapeutic strategies for maintaining glycemic control for the purpose of inducing weight loss and reducing the risk of CVD in adolescents with T1DM (8).

As an insulin-sensitizing agent, metformin can increase glucose uptake variably in the muscle through the amplification of the glucose transporters 4, and the reduction of hepatic glucose production, thus improving tissue sensitivity to insulin (9, 10). It was noted that metformin has been found to help reduce the risk of CVDs and has also been considered as a safe antidiabetic agent in patients with type 2 DM (T2DM) (11). Moreover, guidelines have also made a positive recommendation on the use of adding metformin to insulin therapy in overweight patients with T1DM (12). Certainly, some meta-analyses investigating the effect and safety of metformin in patients with T1DM have also been published (8, 13–15). Among these meta-analyses, two focused on adolescents with T1DM (13, 14). Unfortunately, definitive results were not achieved by them. Moreover, since the publication of two previous meta-analyses, several potentially eligible randomized controlled trials (RCTs) have also been published (16–19). It is necessary to combine all available studies to produce more reliable results for resolving controversial results. Therefore, we performed the current updated meta-analysis to generate a comprehensive assessment regarding the effect and safety of metformin in adolescents with T1DM.

## Material and Methods

The current meta-analysis was conducted in accordance with the Preferred Reporting Items for Systematic Reviews and Meta-Analyses statement (PRISMA) (20, 21). The institutional approval and patients’ informed consent were not required due to the statistical analysis performed based on the published data.

### Information Sources

We systematically searched PubMed, Embase, and the Cochrane Central Registry of Controlled Trials (CENTRAL) from February 2016 to November 2021 for identifying relevant RCTs. We used the following search terms to construct the basic search strategy: metformin, type 1 diabetes mellitus, and random. The detail of the search strategy is summarized in **Table S1**. Furthermore, we also manually searched the reference lists of eligible studies (22). Language and publication status were not restricted in our meta-analysis. Disagreements between two authors were resolved by a third author for resolution.

### Study Selection

Two independent authors assessed the eligibility of each study identified from the literature search and previous meta-analyses in a blinded manner according to the following criteria: (a) participant: adolescents with T1DM and less than 20 years; (b) intervention: metformin regardless of daily dose; (c) control: placebo; (d) outcomes: at least one of the following outcomes: HbA1c level, BMI (kg/m2 or z-score for age and gender), total insulin daily dose (TIDD) (unit/kg/d), hypoglycemia events, diabetes ketoacidosis (DKA) events, and gastrointestinal adverse events (GIAEs); (e) study design: RCTs with the treatment duration of more than 3 months. Studies were excluded if they met the following criteria: abstracts without sufficient data, editorials, and reviews. Disagreements between two authors were resolved by a third author for resolution.

### Data Extraction

Two independent authors extracted the following information from each eligible study using a standardized data extraction sheet: first author, country, publication year, study design, the number of total randomized patients, the percentage of male patients, the number of losing to follow-up, the age of patients, diabetes duration, daily dose of metformin, the duration of treatment, and outcomes. The primary outcome was the HbA1c level, and secondary outcomes included BMI, TIDD, the incidence of hypoglycemia events, DKA events, and GIAEs.

### Quality Assessment

We used the Cochrane risk-of-bias (RoB) assessment tool to assess the risk of bias of each eligible study (23) from the following seven items: random sequence generation, allocation concealment, the blinding of participants and personnel, the blinding of the outcome assessor, incomplete data, selective reporting, and other bias sources. Each item was assessed as “low”, “unclear”, or “high” risk of bias according to the matching level between the reported information of each study and assessment criteria. Disagreements between two authors were resolved by a third author for resolution.

### Statistical Analysis

The weighted mean difference (MD) or risk ratio (RR) with their 95% confidence interval (CI) was used to express the results of continuous variables and dichotomous variables, respectively. We used the methods described by Wan et al. to estimate means and standard deviation (SD) if only medians and interquartile ranges were available (24). Statistical heterogeneity across studies was firstly quantitatively assessed by chi-square (25, 26), and then, we quantified the level of statistical heterogeneity using the I2 statistic (27). Heterogeneity was considered as significant if the I2 value >50%, and then, we selected a random-effects model to calculate pooled results (27). Otherwise, we selected fixed-effects models to conduct a meta-analysis if the I2 value < 50% (28). We performed subgroup analysis to investigate the impact of metformin on the HbA1c level, BMI, and TIDD because data were reported at different time points. Moreover, we also explored the influence of weight on the pooled results using a sensitivity analysis. We used a funnel plot to qualitatively inspect the publication bias (29), and the Egger’s and Begg’s tests were performed to assess the symmetry of a funnel plot (30). All P- values were two sided, and a P-value <0.05 was considered as statistically significant. Meta-analysis was conducted using Review Manager 5.4 (The Nordic Cochrane Centre, the Cochrane Collaboration, Copenhagen, 2014).

## Results

### Search Results

**Figure 1:**
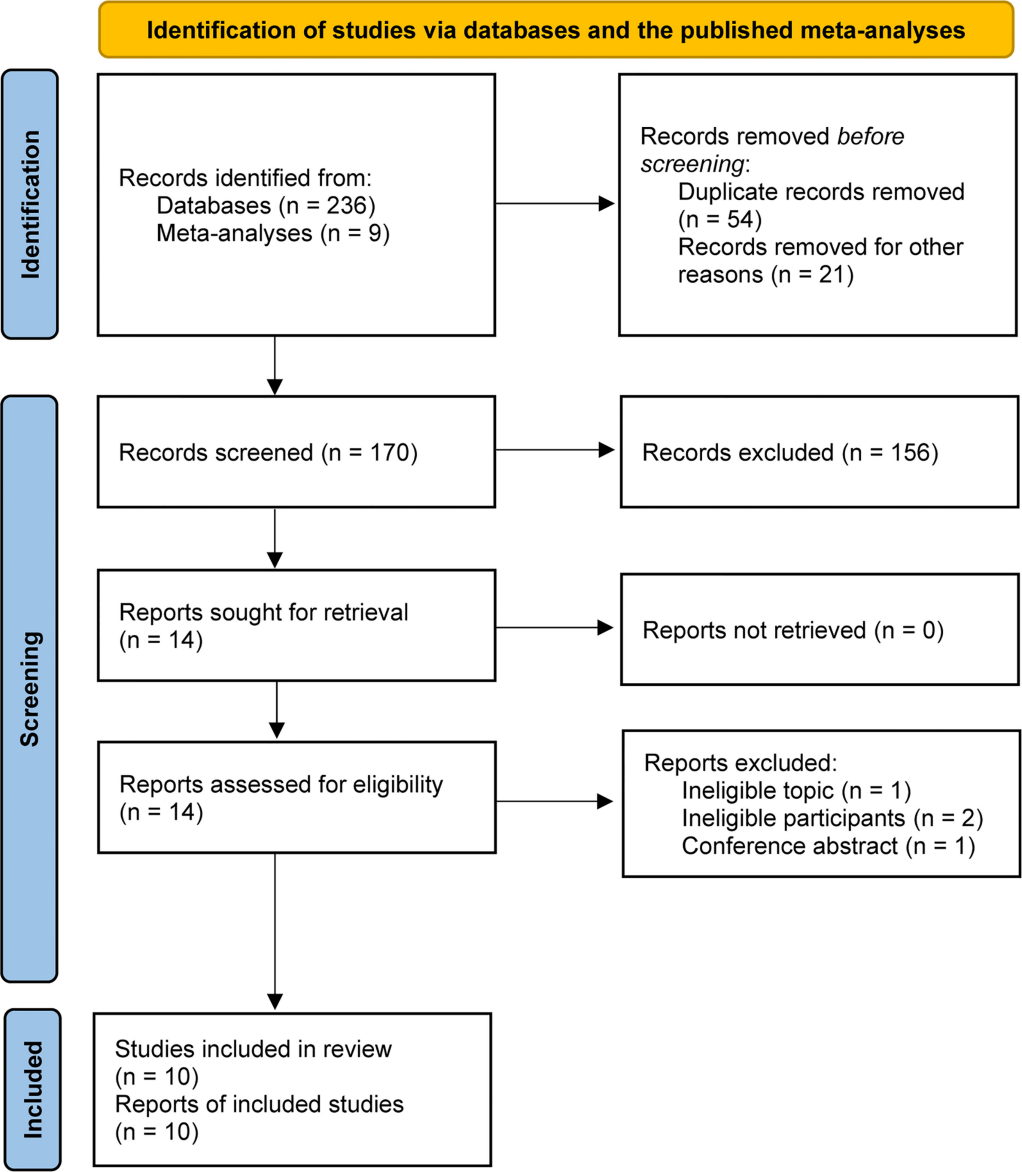
PRISMA flowchart of study retrieval and selection.

A total of 245 relevant studies were identified from the literature search and previous meta-analyses (8, 13–15). We removed 54 duplicates and 21 study protocols based on EndNote software. After checking the titles and abstracts of the remaining studies, 14 studies were retrieved. After evaluating the full text, 10 studies met our selection criteria and were included in the final analysis (16–19, 31–36). The PRISMA flowchart for literature retrieval and selection is displayed in **Figure 1**.

### Characteristics of Studies

The basic characteristics of the included studies are summarized in **Table ​1**. All studies were published between 2003 and 2021. The sample size of each study ranged from 24 to 140, with a total number of 539. Four studies (18, 19, 33, 35) enrolled overweight/obese adolescents, and the remaining studies (16, 17, 31, 32, 34, 36) included general adolescents. The daily dose of metformin was different among studies, ranging from 500 to 2,000 mg. Treatment duration varied from 3 to 12 months. All studies (16–19, 31–36) reported the baseline HbA1c level and total insulin daily dose, seven studies (17, 19, 31, 32, 34–36) reported BMI as kilogram per square meter, and five studies (16, 18, 19, 33, 34) reported BMI as z-score. The baseline data of these indicators is shown in **Table 2**.

**Table 1:** Basic characteristics of 10 eligible studies.

Study	Country	Study design	Target patients	Intervention	Follow-up, months	Total randomized patients, N	Percentage of male patients, %	Lost, N	Age, years	Diabetes duration, years
Anderson, et al., (16)	Australia	SC	General adolescents	Metformin, 2,000 mg daily	12	45	46.67	1	14.0 ± 2.5	5.2 ± 3.6
				Placebo		45	44.44	0	13.3 ± 2.6	5.8 ± 4.1
Bjornstad, et al., (17)	United States	SC	General adolescents	Metformin, 2,000 mg daily	3	25	n.a.	1	17.3 ± 2.3	8.0 ± 3.7
				Placebo		23	n.a.	2	15.9 ± 2.7	7.8 ± 4.4
Codner, et al., (31)	Chile	SC	General adolescents	Metformin, 1,700 mg daily	9	13	n.a.	0	17.7 ± 1.6	9.3 ± 5.1
				Placebo		11	n.a.	1	16.7 ± 1.7	5.5 ± 3.1
Cree-Green, et al., (18)	United States	MC	Overweight/obese adolescents	Metformin, 500–1,000 mg daily	3	19	36.84	0	15.8 ± 2.1	n.a.
				Placebo		18	55.56	1	15.5 ± 2.2	
Hamilton, et al., (32)	Canada	SC	General adolescents	Metformin, 500–2,000 mg daily	3	14	42.86	1	15.9 ± 1.9	9.9 ± 4.4
				Placebo		13	53.85	2	16 ± 1.7	7.0 ± 3.8
Libman, et al., (33)	United States	MC	Overweight/obese adolescents	Metformin, 500–2,000 mg daily	6.5	71	38.03	1	15.4 ± 1.7	7.5 ± 3.6
				Placebo		69	30.43	0	15.1 ± 1.8	6.4 ± 3.0
Nadeau, et al., (34)	United States	SC	General adolescents	Metformin, 1,000–2,000 mg daily	6	40	58.75	12	15.9 ± 1.7	6.7 ± 3.6
				Placebo		40		9	16.0 ± 1.6	6.3 ± 3.5
Nwosu, et al., (35)	United States	SC	Overweight/obese adolescents	Metformin, 1,000 mg daily	9	15	53.33	3	15.0 ± 2.5	5.7 ± 4.4
				Placebo		13	38.46	3	14.5 ± 3.1	5.7 ± 5.0
Sarnblad, et al., (36)	Sweden	MC	General adolescents	Metformin, 500–2,000 mg daily	3	16	31.25	5	17.2 ± 1.7	9.1 ± 5.0
				Placebo		14	28.57	1	16.9 ± 1.4	7.1 ± 3.0
Gourgari, et al., (19)	United States	MC	Obese adolescents	Metformin, 500–2,000 mg daily	6	25	24.00	0	15.6 ± 1.6	n.a.
				Placebo		10	30.00	0	15.5 ± 1.7	

SC, single center; MC, multiple center; n.a., not applicable.

**Table 2:** Baseline values of endpoints for efficacy.

Study	Intervention	HbA1c %	BMI z-score	BMI (kg/m^2^)	Total insulin daily dose, unit/kg/d
Anderson, et al., (16)	Metformin	8.4 ± 3.2	0.9 ± 0.6	n.a.	0.82 ± 0.22
	Placebo	8.8 ± 2.7	0.9 ± 0.5	n.a.	0.85 ± 0.21
Bjornstad, et al., (17)	Metformin	8.7 ± 3.1	n.a.	25.4 ± 4.4	0.8 ± 0.3
	Placebo	8.5 ± 2.9	n.a.	25.3 ± 4.9	0.9 ± 0.3
Codner, et al., (31)	Metformin	10.3 ± 2.3	n.a.	23.7 ± 3.0	1.2 ± 0.4
	Placebo	9.6 ± 1.5	n.a.	26.2 ± 5.5	1.0 ± 0.4
Cree-Green, et al., (18)	Metformin	9.2 ± 1.1	1.9 ± 0.4	n.a.	1.03 ± 0.30
	Placebo	8.4 ± 1.0	1.9 ± 0.3	n.a.	1.03 ± 0.22
Hamilton, et al., (32)	Metformin	9.3 ± 1.4	n.a.	22.8 ± 4.2	1.2 ± 0.3
	Placebo	8.6 ± 0.8	n.a.	25.7 ± 2.9	1.3 ± 0.2
Libman, et al., (33)	Metformin	8.8 ± 0.8	1.6 ± 0.4	n.a.	1.1 ± 0.2
	Placebo	8.8 ± 0.7	1.7 ± 0.3	n.a.	1.1 ± 0.2
Nadeau, et al., (34)	Metformin	9.5 ± 1.3	0.77 ± 0.63	23.5 ± 3.0	1.2 ± 0.2
	Placebo	9.4 ± 1.1	0.81 ± 0.80	24.3 ± 4.1	1.2 ± 0.3
Nwosu, et al., (35)	Metformin	9.3 ± 1.5	n.a.	28.0 ± 5.4	1.1 ± 0.2
	Placebo	8.7 ± 0.4	n.a.	27.7 ± 4.1	1.4 ± 0.5
Sarnblad, et al., (36)	Metformin	9.6 ± 1.0	n.a.	23.5 ± 4.2	1.1 ± 0.3
	Placebo	9.5 ± 1.2	n.a.	23.9 ± 3.05	1.2 ± 0.3
Gourgari, et al., (19)	Metformin	9.1 ± 0.74	1.94 ± 0.36	30.1 ± 4.0	0.96 ± 0.15
	Placebo	8.9 ± 0.30	1.88 ± 0.34	28.7 ± 3.63	1.21 ± 0.26

HbA1c, glycosylated hemoglobin; BMI, body mass index; n.a., not applicable.

### Risk of Bias

**Figure 2:**
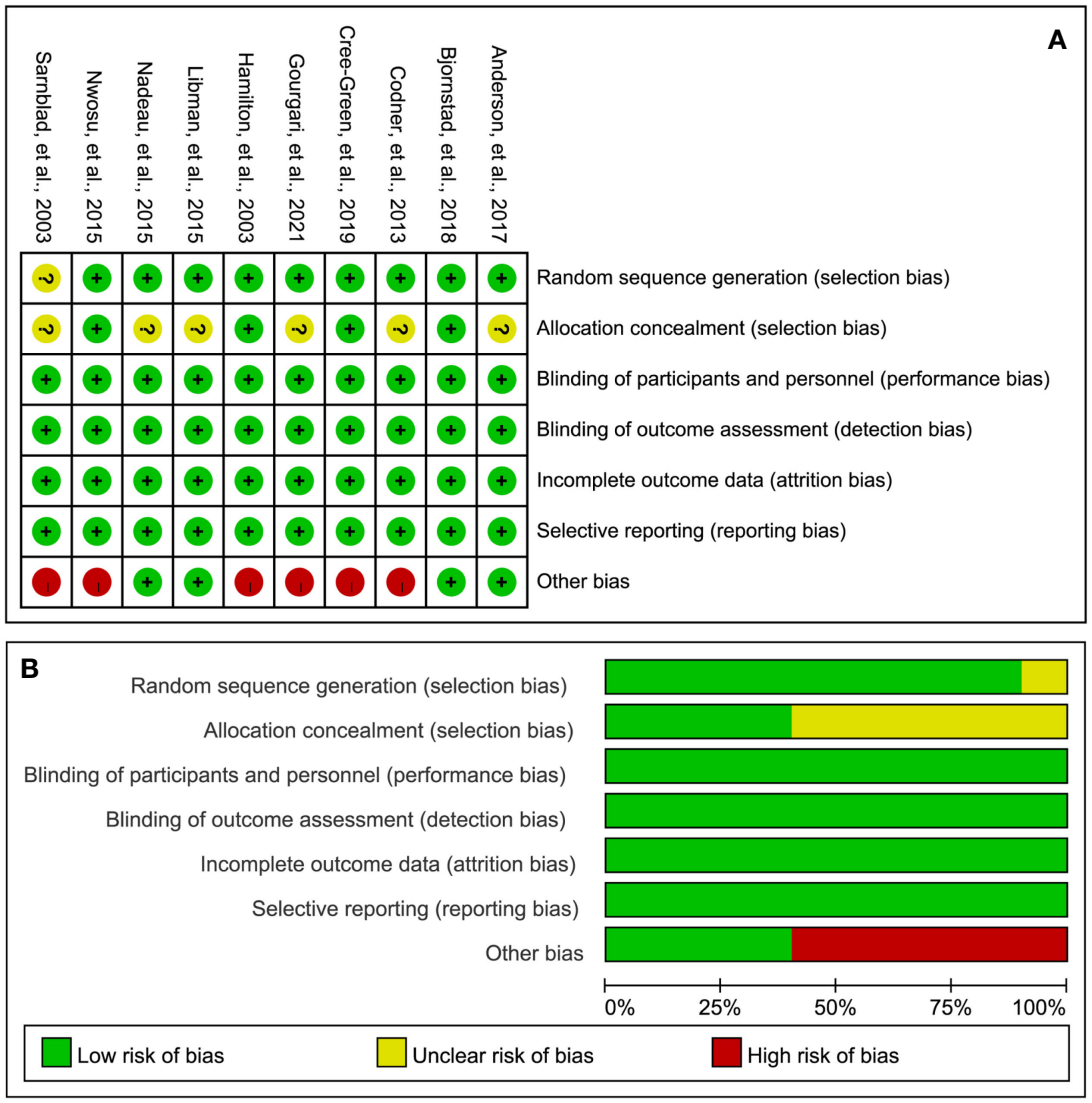
RoB summary **(A)** and graph **(B).** “+”, “?”, and “-” represent “low”, “unclear”, and “high” risk of bias, respectively.

**Figure 2** illustrates the details of the RoB assessment. Overall, one study (17) was judged to be at low overall RoB, three studies (16, 34, 35) were judged to be at moderate overall RoB, and six studies (18, 19, 31–33, 36) were judged to be at high RoB. Of these ten studies, nine studies (16–19, 31–35) appropriately generated a random sequence but only four studies (17, 18, 32, 35) clearly reported the methods of performing allocation concealment. All studies blinded personnel, participants, and the outcome assessor. All studies were also judged to be at low RoB in incomplete outcome data and selective reporting. However, six studies were judged to be at high RoB due to the extremely small sample size.

### HbA1c Level

**Figure 3:**
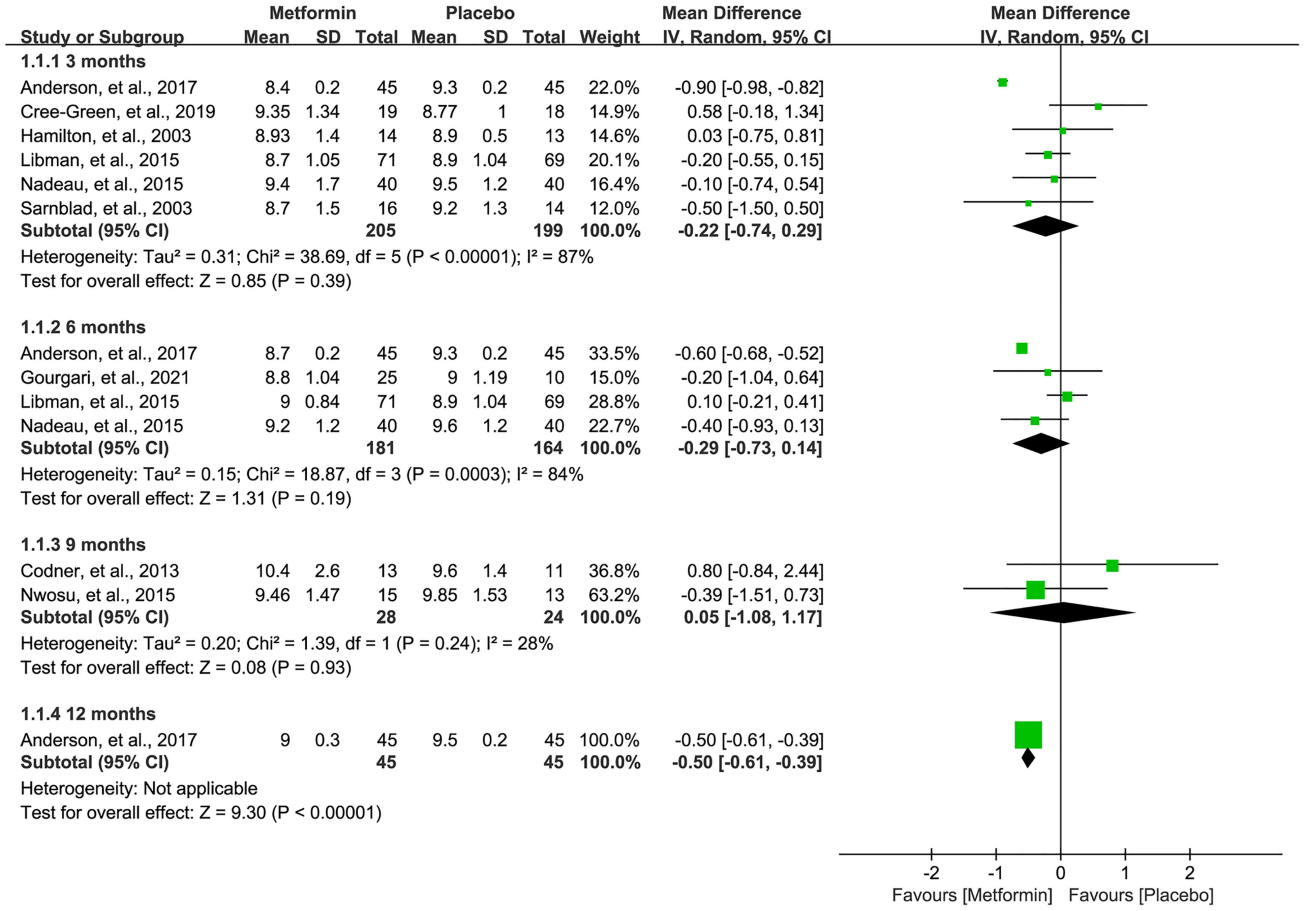
Forest plot of the comparative efficacy between metformin and placebo in terms of HbA1c at different follow-up duration.

A total of nine studies (16, 18, 19, 31–36) reported the HbA1c level after the treatment of metformin. As shown in **Figure 3**, meta-analysis suggested a numerically lower HbA1c level in the metformin group at 3 (MD=-0.22, 95%CI=-0.74 to 0.29, I2 = 87%) and 6 (MD=-0.29, 95%CI=-0.73 to 0.14, I2 = 84%) months, but there was no statistical significance. Meanwhile, the HbA1c level was not statistically significant between the two groups in 9 months (MD=0.05, 95%CI=-1.08 to 1.17, I2 = 28%). However, one study suggested a significantly lower HbA1c level in the metformin group at 12 months after treatment (MD=-0.50, 95%CI=-0.61 to -0.39, I2=N/A).

### BMI

**Figure 4:**
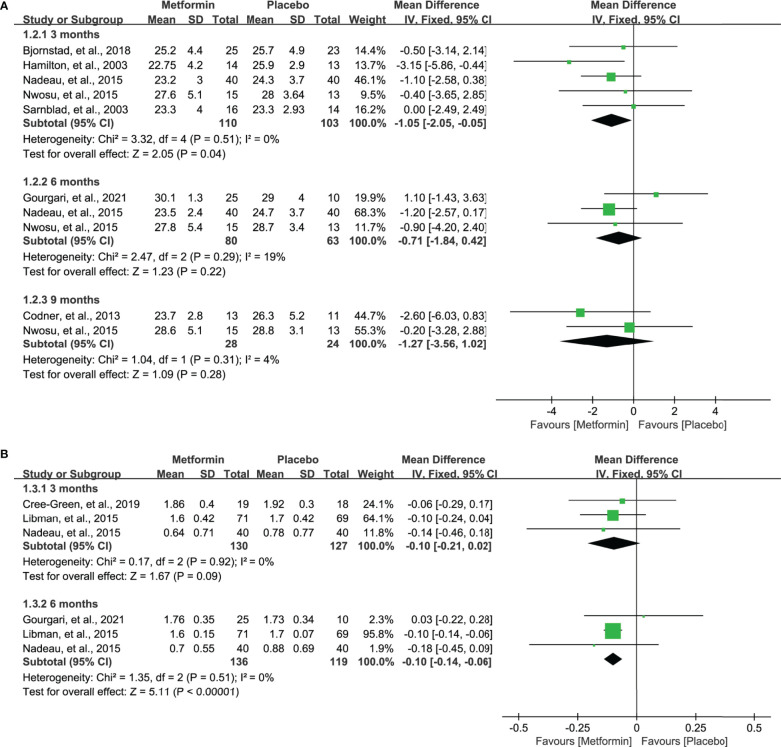
Forest plot of the comparative efficacy between metformin and placebo in terms of BMI at different follow-up duration. **(A)** BMI (kg/m^2^) and **(B)** BMI z-score.

Nine studie s (17–19, 31–36) reported the data of BMI after the treatment of metformin. However, seven studies (17, 19, 31, 32, 34–36) reported the BMI (kg/m2) and four studies (18, 19, 33, 34) reported the BMI z-score. Meta-analysis suggested a significantly lower BMI (kg/m2) at 3 months in patients receiving metformin (MD=-1.05, 95%CI=-2.05 to -0.05, I2 = 0%), as shown in **Figure 4A**. However, no statistical difference was identified at 6 and 9 months, although a numerically lower BMI was achieved. Moreover, as shown in **Figure 4B**, metformin significantly lowered the BMI z-score in 6 months (MD=-0.10, 95%CI=-0.14 to -0.06, I2 = 0%) and also numerically decreased the BMI z-score in 3 months (MD=-0.10, 95%CI=-0.21 to 0.02, I2 = 0%).

### TIDD

**Figure 5:**
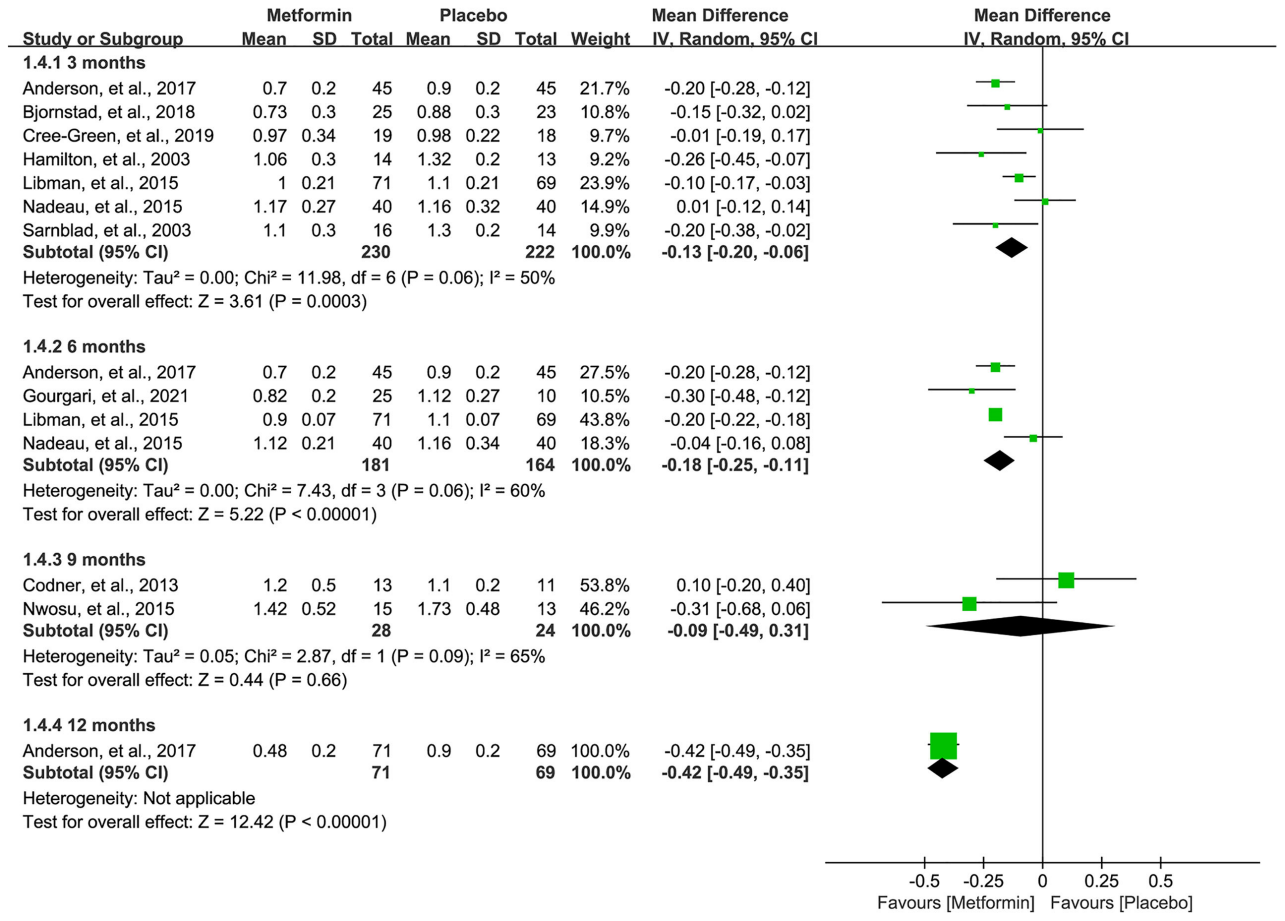
Forest plot of the comparative efficacy between metformin and placebo in terms of total insulin daily dose at different follow-up duration.

All eligible studies (16–19, 31–36) reported the data of TIDD, and meta-analysis suggested a significantly lower TIDD in 3 (MD=-0.13, 95%CI=-0.20 to -0.06, I2 = 50%), 6 (MD=-0.18, 95%CI=-0.25 to -0.11, I2 = 60%), and 12 (MD=-0.42, 95%CI=-0.49 to -0.35, I2=N/A) months in patients receiving metformin. However, no statistical difference was achieved, although metformin numerically lowered the TIDD in 9 months compared with placebo. Pooled results are shown in **Figure 5**.

### Adverse Events

**Figure 6:**
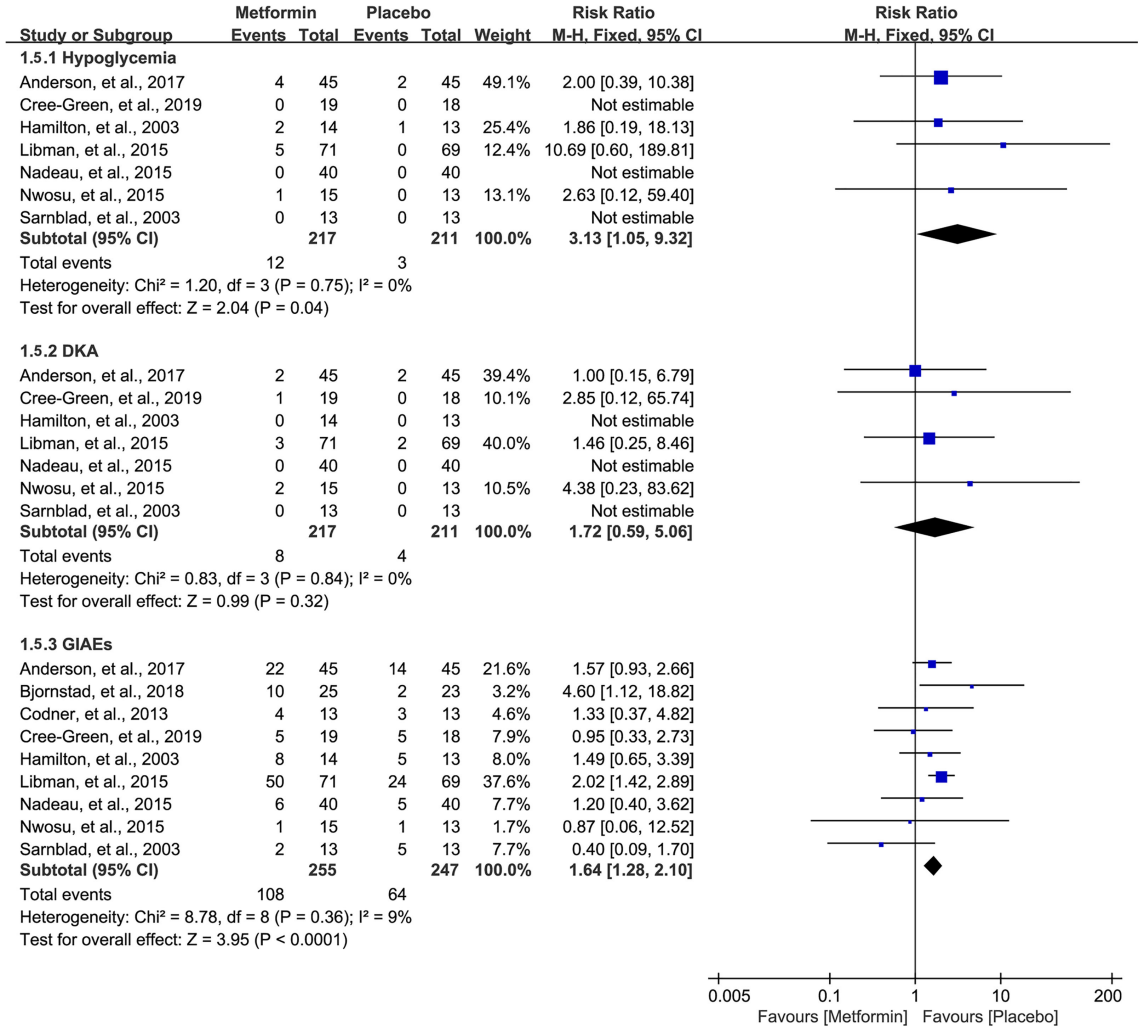
Forest plot of the comparative safety between metformin and placebo in terms of adverse events during the treatment.

Data on hypoglycemia, DKA, and GIAEs were available in seven (16, 18, 32–36), seven (16, 18, 32–36), and nine studies (16–18, 31–36), respectively. The pooled result showed that metformin was associated with a higher incidence of hypoglycemia (RR=3.13, 95%CI=1.05 to 9.32, I2 = 0%) and GIAEs (RR=1.64, 95%CI=1.28 to 2.10, I2 = 9%) (**Figure 6**). However, no statistical difference was detected for the incidence of DKA, although a numerically higher incidence of DKA occurred in the metformin group (RR=1.72, 95%CI=0.59 to 5.06, I2 = 0%) (**Figure 6**).

### Sensitivity Analysis

Targeted participants were diverse in the included studies, including general adolescents and overweight/obese adolescents. We therefore performed a series of sensitivity analyses to examine the robustness of the pooled results. For HbA1c, BMI (kg/m2), and TIDD at 3, 6, and 9 months, sensitivity analyses were conducted through excluding those studies in which only overweight/obese adolescents were included, and all results were not significantly changed after performing sensitivity analysis. For the BMI z-score, we performed sensitivity analyses through excluding those studies in which only general adolescents were included, and pooled results were also not significantly changed. The results of all sensitivity analyses are shown in **Table S2**, suggesting that all pooled results in our meta-analysis were robust.

### Publication Bias

**Figure 7:**
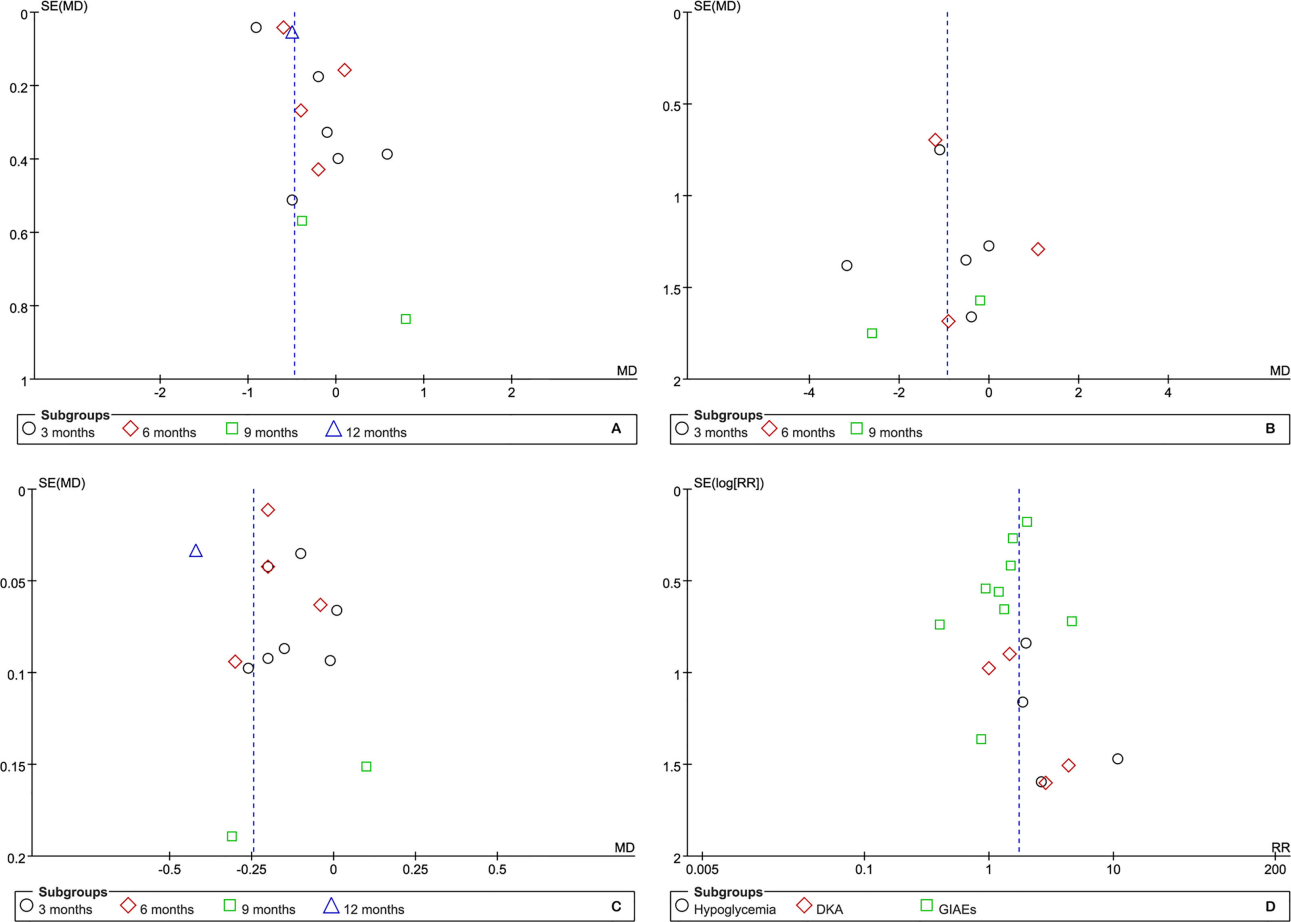
Funnel plot of publication bias for the comparative efficacy and safety.

Although the number of eligible studies for each outcome was not more than 10, we still performed publication bias for those outcomes of which a relatively adequate number of the included studies was accumulated. According to the results of funnel plots, as shown in **Figure 7**, publication bias may have a negative impact on the pooled results of HbA1c but have no impact on other outcomes. Furthermore, Egger’s and Begg’s tests supported the presence of publication bias in HbA1c at 3 months (t=3.89 and p=0.018 for Egger’s test, z=0.00 and p=1.00 for Begg’s test) and the absence of publication bias in BMI (kg/m2) (t=-0.08 and p=0.938 for Egger’s test, z=-0.24 and p=1.00 for Begg’s test) and TIDD (t=-0.02 and p=0.983 for Egger’s test, z=0.30 and p=0.764 for Begg’s test) at 3 months, hypoglycemia (t=1.05 and p=0.405 for Egger’s test, z=1.020 and p=0.308 for Begg’s test), DKA (t=2.34 and p=0.145 for Egger’s test, z=0.34 and p=0.734 for Begg’s test), and GIAEs (t=-1.13 and p=0.294 for Egger’s test, z=1.36 and p=0.175 for Begg’s test).

## Discussion

### Main Findings

Metformin is one of the most widely used antidiabetic agents for improving cardiovascular prognosis in patients with T2DM (37) and is also recommended for patients with T1DM recently (12). Although previous two meta-analyses (13, 14) have been conducted to investigate the effect and safety of adding metformin to insulin therapy for adolescents with T1DM, no consensus has been achieved. In this updated meta-analysis, ten eligible studies involving 539 adolescents with T1DM were included in the final analysis, and pooled results suggested a significant reduction in the HbA1c level at 12 months, BMI (kg/m2) at 3 months, and BMI z-score at 6 months, as well as TIDD at 3, 6, and 12 months, and a significant increase in the risk of hypoglycemia events and GIAEs during the metformin treatment. For other outcomes on therapeutic efficacy, numerical benefits were also detected in those participants receiving metformin, although no statistical significance was achieved.

### Comparison With Previous Meta-Analyses

Actually, up to now, two meta-analyses (13, 14) have been published to investigate the therapeutic efficacy and safety of adding metformin to insulin therapy for adolescents with T1DM. In 2016, Liu et al. (14) included five eligible studies in their meta-analysis, and pooled results suggested significant changes in HbA1c levels in the general adolescents and significant changes in BMI in overweight/obese adolescents. Meanwhile, metformin was associated with a higher risk of adverse events. It was noted that this meta-analysis combined the data reported in the different time points to calculate the pooled results, which significantly increased the risk of false-positive findings, although it conducted separate analyses according to target participants. Meanwhile, this meta-analysis also combined the BMI z-score and BMI (kg/m2) as the individual endpoint. In 2017, Khalifah et al. (13) also performed a meta-analysis to investigate the effect of adding metformin to insulin therapy for T1DM children. In this meta-analysis, 6 studies involving 325 participants were included for analysis and pooled results suggested that the addition of metformin significantly decreased TIDD and also decreased the BMI (kg/m2) and BMI z-score. Unfortunately, in this meta-analysis, the authors also simply combined the data at different time points to calculate the pooled results. Moreover, as one of the most common adverse events, GIAEs were not assessed by this meta-analysis. There were another two meta-analyses (8, 15) that also investigated the role of metformin in general patients with T1DM; however a subgroup analysis of adolescents was not conducted. Compared with previous meta-analyses, the current updated meta-analysis included more eligible studies (10 eligible studies involving 539 adolescents) to generate more robust and reliable findings. More importantly, we conducted subgroup analysis to separately investigate the effect of metformin treatment for adolescents with T1DM at different treatment duration, which greatly decreased the risk of introducing bias. Moreover, we also conducted sensitivity analysis to examine the robustness of pooled results through differentiating targeted participants into general adolescents or overweight/obese adolescents (14), which further confirmed the reliability of our findings.

### Strengths and Limitations

The present updated meta-analysis has several strengths as follows: (a) we identified relevant studies through simultaneously conducting a highly sensitive literature retrieval strategy and checking the reference lists of the published meta-analyses; (b) we used the Cochrane RoB assessment tool to quantify the level of methodological quality of the included studies; (c) a series of sensitivity analyses have been conducted to examine the robustness of the pooled results, suggesting the reliability of findings from our updated meta-analysis; and (d) we firstly used a funnel plot to qualitatively inspect the publication bias and then conducted Egger’s test and Begg’s tests to quantitatively examine the risk of asymmetry of the funnel plot, which greatly increased the statistical power of detecting the publication bias.

Nevertheless, our updated meta-analysis has many limitations as follows: (a) most included studies (60.0%) enrolled an extremely insufficient sample size (<20 in each group), which significantly increased the risk of overestimating the therapeutic efficacy of metformin administration on adolescents with T1DM; (b) only four studies clearly described the details of allocation concealment, although nine studies conducted an appropriate random sequence; (c) most eligible studies (60.0%) were performed in a single center and in European countries, which may reduce the generality of our findings into different clinical settings; (d) variations were detected in some aspects such as diabetes duration, the percentage of male participants, and the mean age of participants, which may confound the pooled results because subgroup or sensitivity analysis was not conducted due to limited data; and (e) T1DM has been found to be associated with an increased risk of CVD; however the effect of metformin administration on CVD was not investigated in this meta-analysis.

## Conclusion

Our meta-analysis indicated that metformin administration may decrease the BMI (kg/m^2^) at 3 months; BMI z-score at 6 months; and TIDD at 3, 6, and 12 months and increase the risk of hypoglycemia events and GIAEs in adolescents with T1DM. Metformin may be recommended as an adjunct to insulin therapy for adolescents with T1DM; however, future studies are required to determine the optimal dose and duration of metformin administration.

## Data Availability Statement

The original contributions presented in the study are included in the article/**Supplementary Material**. Further inquiries can be directed to the corresponding authors.

## Author Contributions

YL, HC, and HuL carried out the studies, participated in collecting data, and drafted the manuscript. YL and LL performed the statistical analysis and participated in its design. HoL and JW participated in the acquisition, analysis, or interpretation of data and drafted the manuscript. All authors read and approved the final manuscript.

## Conflict of Interest

The authors declare that the research was conducted in the absence of any commercial or financial relationships that could be construed as a potential conflict of interest.

## Publisher’s Note

All claims expressed in this article are solely those of the authors and do not necessarily represent those of their affiliated organizations, or those of the publisher, the editors and the reviewers. Any product that may be evaluated in this article, or claim that may be made by its manufacturer, is not guaranteed or endorsed by the publisher.

## Supplementary Material

Supplementary Table 1 Search queries of all target databases.

Supplementary Table 2 Sensitivity analysis of HbA1c, BMI, and TIDD at 3, 6 and 9 months. HbAc, glycalyted hemoglobin; BMI, body mass index; TIDD, total insulin daily dose.
