# Supplementary material for: Effect and Safety of Adding Metformin to Insulin Therapy in Treating Adolescents With Type 1 Diabetes Mellitus: An Updated Meta-Analysis of 10 Randomized Controlled Trials
Source: Front Endocrinol (Lausanne). 2022 May 30;13:878585. doi: 10.3389/fendo.2022.878585 (PMC9190285; doi:10.3389/fendo.2022.878585)
Supplement: Supplementary Table 1 — Search queries of all target databases. [file Table_1.docx]

**PubMed**

| Search number | Query | Filters | Results | Time |
| --- | --- | --- | --- | --- |
| 9 | #3 and #6 and #7 | from 2016/1/1 - 2021/11/30 | 49 | 4:53:03 |
| 8 | #3 and #6 and #7 | | 145 | 4:51:10 |
| 7 | random* |  | 1,517,281 | 4:50:55 |
| 6 | #4 or #5 | | 25,790 | 4:50:16 |
| 5 | (((((Dimethylbiguanidine[Title/Abstract]) OR (Dimethylguanylguanidine[Title/Abstract])) OR (Glucophage[Title/Abstract])) OR (Metformin Hydrochloride[Title/Abstract])) OR (Metformin[Title/Abstract])) OR (melbine[Title/Abstract]) | | 23,917 | 4:49:58 |
| 4 | "Metformin"[Mesh] | | 15,471 | 4:48:38 |
| 3 | #1 or #2 | | 103,624 | 4:47:52 |
| 2 | ((((((((((((Insulin-Dependent Diabetes Mellitus[Title/Abstract]) OR (Juvenile-Onset Diabetes Mellitus[Title/Abstract])) OR (IDDM[Title/Abstract])) OR (Juvenile-Onset Diabetes[Title/Abstract])) OR (Juvenile Onset Diabetes[Title/Abstract])) OR (Sudden-Onset Diabetes Mellitus[Title/Abstract])) OR (Type 1 Diabetes Mellitus[Title/Abstract])) OR (Insulin-Dependent Diabetes Mellitus 1[Title/Abstract])) OR (Insulin Dependent Diabetes Mellitus 1[Title/Abstract])) OR (Type 1 Diabetes[Title/Abstract])) OR (Autoimmune Diabetes[Title/Abstract])) OR (Brittle Diabetes Mellitus[Title/Abstract])) OR (Ketosis-Prone Diabetes Mellitus[Title/Abstract]) | | 64,832 | 4:47:38 |
| 1 | "Diabetes Mellitus, Type 1"[Mesh] | | 80,702 | 4:45:55 |

**Embase** <1974 to 2021 December>

1 (Insulin-Dependent Diabetes Mellitus or Juvenile-Onset Diabetes Mellitus or IDDM or Juvenile-Onset Diabetes or Juvenile Onset Diabetes or Sudden-Onset Diabetes Mellitus or Type 1 Diabetes Mellitus or Insulin-Dependent Diabetes Mellitus 1 or Insulin Dependent Diabetes Mellitus 1 or Type 1 Diabetes or Autoimmune Diabetes or Brittle Diabetes Mellitus or Ketosis-Prone Diabetes Mellitus).tw. 92519

2 Diabetes Mellitus, Type 1.sh. 814

3 1 or 2 92905

4 (Dimethylbiguanidine or Dimethylguanylguanidine or Glucophage or Metformin Hydrochloride or Metformin or melbine).tw. 41194

5 Metformin.sh. 72617

6 4 or 5 75290

7 random*.af. 1969666

8 3 and 6 and 7 340

9 limit 8 to (embase and yr="2016 - 2021") 105

**EBM Reviews - Cochrane Central Register of Controlled Trials <November 2021>**

1 (Insulin-Dependent Diabetes Mellitus or Juvenile-Onset Diabetes Mellitus or IDDM or Juvenile-Onset Diabetes or Juvenile Onset Diabetes or Sudden-Onset Diabetes Mellitus or Type 1 Diabetes Mellitus or Insulin-Dependent Diabetes Mellitus 1 or Insulin Dependent Diabetes Mellitus 1 or Type 1 Diabetes or Autoimmune Diabetes or Brittle Diabetes Mellitus or Ketosis-Prone Diabetes Mellitus).tw. 9693

2 Diabetes Mellitus, Type 1.sh. 5868

3 1 or 2 11537

4 (Dimethylbiguanidine or Dimethylguanylguanidine or Glucophage or Metformin Hydrochloride or Metformin or melbine).tw. 11024

5 Metformin.sh. 4328

6 4 or 5 11351

7 random*.af. 1473121

8 3 and 6 and 7 216

9 limit 8 to yr="2016 - 2021" 82
