# Supplementary material for: Effect and Safety of Adding Metformin to Insulin Therapy in Treating Adolescents With Type 1 Diabetes Mellitus: An Updated Meta-Analysis of 10 Randomized Controlled Trials
Source: Front Endocrinol (Lausanne). 2022 May 30;13:878585. doi: 10.3389/fendo.2022.878585 (PMC9190285; doi:10.3389/fendo.2022.878585)
Supplement: Supplementary Table 2 — Sensitivity analysis of HbA1c, BMI, and TIDD at 3, 6 and 9 months. HbAc, glycalyted hemoglobin; BMI, body mass index; TIDD, total insulin daily dose. [file Table_2.docx]

**Table S2.** Sensitivity analysis

| **Outcome** | **I2** | **P-value for heterogeneity** | **MD, 95%CI** | **Z-value** | **P-value** |
| --- | --- | --- | --- | --- | --- |
| **HbA1c, %**, after excluding studies including overweight/obese adolescents. | | | | | |
| 3 months | 74% | 0.009 | -0.43 (-1.00, 0.13) | 1.50 | 0.13 |
| 6 months | 89% | 0.0001 | -0.31 (-0.81, 0.19) | 1.21 | 0.23 |
| 9 months | n.a. | n.a. | 0.80 (-0.84, 2.44) | 0.96 | 0.34 |
| **BMI in kg/m2**, after excluding studies including overweight/obese adolescents. | | | | | |
| 3 months | 5% | 0.37 | -1.12 (-2.21, -0.03) | 2.01 | 0.04 |
| 6 months | 0% | 0.87 | -1.16 (-2.42, 0.11) | 1.79 | 0.07 |
| 9 months | n.a. | n.a. | -2.60 (-6.03, 0.83) | 1.49 | 0.14 |
| **BMI z-score,** after excluding studies including general adolescents. | | | | | |
| 3 months | 0% | 0.77 | -0.09 (-0.21, 0.03) | 1.47 | 0.14 |
| 6 months | 0% | 0.32 | -0.10 (-0.14, -0.06) | 4.82 | <0.001 |
| **TIDD, unit/kg/day**, after excluding studies including overweight/obese adolescents. | | | | | |
| 3 months | 54% | 0.07 | -0.16 (-0.22, -0.10) | 5.35 | <0.001 |
| 6 months | 77% | 0.04 | -0.15 (-0.22, -0.08) | 4.30 | <0.001 |
| 9 months | n.a. | n.a. | 0.10 (-0.20, 0.40) | 0.66 | 0.51 |

HbA1c, glycalyted hemoglobin; BMI, body mass index; TIDD, total insulin daily dose; n.a., not applicable; MD, mean difference; CI, confidence interval.
